# Exploring the efficacy and safety of Yu-Ping-Feng powder with variation against allergic rhinitis: a randomized, double-blind, placebo-controlled trial

**DOI:** 10.1186/s13020-025-01120-2

**Published:** 2025-05-26

**Authors:** Zhi-Xiu Lin, Tin Muk Ho, Yan-Fang Xian, Kam Leung Chan, Qing-Qing Xu, Cho Wing Lo, Justin Che Yuen Wu, Kam Lun Hon, Sin Bond Leung, Chon Pin Chia, Chi Him Sum, Tak Yee Chow, Pui Kuan Cheong, Jessica Yuet Ling Ching, Hongwei Zhang, Ka Chun Leung, Wai Ling Lin

**Affiliations:** 1 School of Chinese Medicine, Faculty of Medicine, The Chinese University of Hong Kong, Shatin, N.T., Hong Kong, China; 2 Hong Kong Institute of Integrative Medicine, Faculty of Medicine, The Chinese University of Hong Kong, Shatin, N.T., Hong Kong, China; 3 The Chinese University of Hong Kong Chinese Medicine Specialty Clinic Cum Clinical Teaching and Research Centre, Faculty of Medicine, The Chinese University of Hong Kong, Shatin, N.T., Hong Kong, China; 4 Department of Medicine and Therapeutics, Faculty of Medicine, The Chinese University of Hong Kong, Shatin, N.T., Hong Kong, China; 5 Department of Paediatrics, Faculty of Medicine, The Chinese University of Hong Kong, Shatin, N.T., Hong Kong, China

**Keywords:** Allergic rhinitis, Yu-Ping-Feng powder with variation (YPV), RCT

## Abstract

**Background:**

Allergic rhinitis (AR) is a common allergic condition characterized by frequent sneezing, nasal congestion, nasal itching and rhinorrhea. Chinese medicine formula Yu-Ping-Feng Powder with Variation (YPV) is an empirical formula modified from an ancient Chinese medicine formula named Yu-Ping-Feng Powder, which is widely used for the treatment of allergic diseases such as AR and asthma.

**Purpose:**

To evaluate the efficacy and safety of YPV on AR patients with the lung and spleen Qi deficiency type using a randomized, double-blind, placebo-controlled clinical trial (RCT).

**Study design and methods:**

Between April 2022 and June 2023, a total of 58 participants were recruited and randomly allocated to receive either YPV (n = 29) or placebo (n = 29) for 8 consecutive weeks. The changes of the Total Nasal Symptom Score (TNSS), and the Rhinoconjunctivitis Quality of Life Questionnaire (RQLQ) or the Paediatric Allergic Disease Quality of Life Questionnaire (PADQLQ) scores at week 8 were used as the primary outcomes. The secondary outcomes included (1) the change of TNSS at weeks 4, 12 and 16; (2) the RQLQ or the PADQLQ scores at weeks 4, 12 and 16; (3) the change of frequency of AR episodes and their severity Visual Analog Scale (VAS) at weeks 4, 8, 12 and 16; (4) the changes of the gut microbiota composition in stool samples at week 8; and (5) adverse events related to the study treatment.

**Results:**

YPV treatment could significantly improve the RQLQ score in AR patients at weeks 4, 8, 12, and 16 (*p* = 0.05, *p* = 0.04, *p* = 0.04 and *p* = 0.03, respectively), when compared with the placebo treatment. However, it did not improve the TNSS score at week 8 in AR patients when compared with the placebo group. In addition, YPV treatment could reduce the VAS score in AR patients at weeks 12 and 16 when compared with the placebo group, although the reductions were not statistically significant (*p* = 0.06 and *p* = 0.08, respectively). Importantly, no overt adverse effects were observed in both YPV and placebo groups.

**Conclusion:**

YPV was well-tolerated and could effectively ameliorate multiple symptoms of AR and improve the quality of life of AR patients after 8-week treatment.

**Trial registration**

ClinicalTrials.gov, NCT04976023. Registered 26 July 2021, https://clinicaltrials.gov/study/NCT04976023?cond=The%20Effects%20of%20Using%20Yupingfeng%20Powder%20with%20Variation%20for%20the%20Treatment%20of%20Allergic%20Rhinitis&rank=2

## Introduction

Allergic rhinitis (AR), a worldwide health problem, not only severely affects the sleep pattern and quality of life of the patients and their work and school performance, but also imposes heavy economic burden to the patients and their families [1–3]. The typical clinical symptoms of AR include frequent sneezing, nasal itching and congestion, and rhinorrhea [4]. The prevalence of AR has been increasing in the past decade. It has been reported that more than 50% of children and 5–40% of adults are affected by AR in some countries [5]. As for the medical treatment, intranasal corticosteroids are considered to be the most effective medication for controlling the symptoms of AR [6]. Currently, AR is commonly treated with corticosteroids, H1 anti-histamines, leukotriene receptor antagonists and chromones [7]. However, problems such as drug intolerance, and lack of efficacy are often associated with these therapeutics [8, 9].

Given that multiple factors are involved in AR pathogenesis, multi-target drug development is now perceived as a more promising therapeutic strategy for AR treatment. Owing to the multiple-target, multi-pharmacology and low toxicity characteristics, Chinese medicine (CM) has attracted increasing attention in recent years for AR. AR is induced by Qi deficiency of the lung and the spleen in most cases, and the external wind pathogen is invaded to the nasal orifices, inducing the symptoms of AR in CM [10, 11]. Thus, the major treatment principle for AR is tonifying the Qi of lung and spleen in CM. Yu-Ping-Feng Powder, composed of three Chinese herbal medicines (CHMs) including the dried root of *Astragalus membranaceus* (Fisch.) Bunge (Huangqi in Chinese), the dried root of *Atractylodes macrocephala* Koidz. (Baizhu in Chinese) and the dried root of *Saposhnikovia divaricate* (Turcz.) Schischk (Fangfeng in Chinese) at the ratio of 2:2:1, was first recorded in the Danxi’s Mastery of Medicine (Dan-Xi-Xin-Fa in Chinese), which was written by a famous Chinese medicine physician Zhu Danxi in the Yuan dynasty (AD 1279–1368). Yu-Ping-Feng Powder is widely used for the treatment of allergic diseases such as AR and asthma in CM practice [12].

Clinical studies demonstrated that Yu-Ping-Feng Powder could ameliorate the topical symptoms of AR such as sneezing and nasal itching [13, 14], and also improve the quality of life of AR patients [13] via inhibiting the release of immunoglobulin E (IgE) and interleukin-4 (IL-4) [14]. Other clinical studies also found that Yu-Ping-Feng Powder could enhance the anti-allergic ability [13] and reduce the body’s hyper-reaction [15]. Yu-Ping-Feng Powder with Variation (YPV), an empirical formula commonly prescribed by the team members in clinical practice to treat AR, is formed by adding the dried flower of *Magnolia denudata* Desr (Xinyihua in Chinese), the dried fruit of *Xanthium sibiricum* Patrin ex Widder (Cangerzi in Chinese), the dried fruiting body of *Ganoderma lucidum* (Leyss. ex Fr.) Karst (Chilingzhi in Chinese), the dried root of Platycodon grandiflorus (Jacq.) A. DC.) (Jiegeng in Chinese), the dried fossil of *Periploca forrestii* Schltr (Duanlonggu in Chinese), the dried shell of *Ostrea gigas* Thunbery (Duanmuli in Chinese), the dried fruit of *Terminalia chebula* Retz (Hezi in Chinese), the dried root of *Glehnia littoralis* Fr. Schmidtex Miq. (Beishashen in Chinese), The dried powder of *Cordyceps sinensis* (Berk.) Sacc. (Chongcaojunsifen in Chinese), the dried fruit of *Dictamnus dasycarpus* Turcz. (Baixianpi in Chinese) into the original Yu-Ping-Feng Powder. Among of them, Magnoliae Flos is a common herb used for treating the symptoms of allergic rhinitis, sinusitis and headache [16–18]. Xanthii Fructu*s* is traditionally used to reduce nasal obstruction, nasal discharge, rhinitis with nasal discharge, and rhinitis with muscular spasms caused by wind and dampness [19]. Magnoliae Flo*s*, processed Xanthii Fructus*,* Ganoderma, and Platycodonis Radix are added to the original Yu-Ping-Feng Powder to further reduce the nasal congestion of AR. Processed Os Draconis, processed Ostreae Concha, Dictamni Cortex and Chebulae Fructus are able to reduce sneeze and nasal discharge, two major clinical symptoms of AR patients. Ganoderma and Cordyceps sinesis powder are well-known for their effect in enhancing the immunity of the body, while Glehniae Radix consolidates the lung qi, and strengthens the effects of the original Yu-Ping-Feng Powder [20, 21]. Allergy plays a very important role in the development of AR. Our previous preclinical study revealed that Dictamni Cortex possesses significant anti-allergic effect in an atopic dermatitis animal model [22]. Although YPV is observed to exert good therapeutic effect in clinical practice for improving the clinical symptoms of AR patients, sound clinical evidence concerning the efficacy and safety of YPV on AR is still lacking. In this study, we performed a randomized, double-blind, placebo-controlled clinical trial to measure the efficacy and safety of YPV on AR patients with the lung and spleen Qi deficiency syndrome type.

## Materials and methods

### Study design

This is a multi-centre, randomized, double-blind, placebo-controlled trial conducted between April 2022 and June 2023. The trial protocol was published previously [22]. The ethics approval for the trial was granted by The Joint Chinese University of Hong Kong-Hospital Authority New Territories East Cluster Clinical Research Ethics Committee (CREC Ref. No.:2021.057-T) prior to the trial commencement. The trial was registered on ClinicalTrials.gov on 26 July 2021 (NCT04976023), and was conducted according to the Declaration of Helsinki and Guideline for Good Clinical Practice of the International Council for Harmonization of Technical Requirements for Pharmaceuticals for Human Use. All subjects agreed to provide written informed consent.

### Participants

Inclusion criteria were those patients aged 5 or above with (1) the deficiency of the lung and spleen Qi syndrome type, which were determined by Chinese Medicine Practitioners (CMP) (A checklist of 13 Traditional Chinese medicine (TCM) symptoms were employed for the determination of such syndrome type. Subjects with 2 or more of the listed symptoms were classified as the deficiency of the lung and spleen Qi type); and (2) at least 2 or more allergic symptoms such as sneezing, rhinorrhea, nasal itching and nasal obstruction for a cumulative period greater than 1 h per day, and these symptoms may be accompanied by itchy and red eyes and tears. Exclusion criteria were: (1) known chronic disease such as rhinosinusitis, asthma, nasal polyposis; (2) known severe medical conditions, such as liver or renal dysfunction, cerebrovascular diseases, diabetes mellitus, blood system diseases, and cancers; (3) concomitant use of steroids, anticoagulants, nonsteroidal anti-inflammatory drugs (NSAIDs), and immunotherapy within the past month; (4) impaired hematological profile and liver/renal function that exceeds the upper limit of the reference value by 2 times; (5) known alcohol and/or drug abuse; (6) known allergic history to any CHMs; and (7) known pregnant or lactating.

### Randomization and blinding

Participants were randomly assigned to treatment groups by the random allocation list generated using a computer program to receive either YPV granule or placebo granule in a 1:1 ratio for 8 consecutive weeks. The randomization codes for study medications (YPV or placebo) were generated using a computer. The codes could not be revealed but kept in opaque sealed envelopes with consecutive study numbers. Each sealed envelope was made in duplicates for randomization and unblinding respectively. The envelopes for unblinding remained sealed if there was no need for unblinding. Concealment of allocation was ascertained by an independent research staff member, and identically designed treatment packs were used for study drugs. Attending Chinese medicine practitioners, investigators performing assessments in clinic visits and study participants were blinded to the group allocation until study completion. The dosages were prepared according to the CMP’s instruction. Allocation concealment was conducted using sequentially numbered opaque and sealed envelopes prepared before the trial by an independent staff member not involved in the study. In addition, the CMP investigators, study participants and outcome assessors involved in this study were all kept blind to the allocated intervention.

### Intervention

The eligible participants were stochastically divided into two groups to receive either placebo granules or YPV granules, respectively, for 8 consecutive weeks. The daily dosage of YPV consists of the following CHM granules: Astragali Radix 4 g, processed Ostreae Concha 3.0 g, processed Os Draconis 3.0 g, Ganoderma 2.4 g, Glehniae Radix 2.0 g, Atractylodis Macrocephalae Rhizoma 6.6 g, Magnoliae Flos 2.0 g, Platycodonis Radix 2.0 g, Dictamni Cortex 2.0 g, processed Xanthii Fructus 2.0 g*,* Chebulae Fructus 2.0 g, Saposhnikoviae Radix 2.0 g, Cordyceps sinesis powder 3.0 g. It is worth noting that 1.0 g of the granule is equal to 5.0 g of the dry herb, except for Atractylodis Macrocephalae Rhizoma, which is equal to 3.0 g of the dry herb. Also, since Cordyceps sinesis is made in ground powder form, the concentration ratio of this ingredient is 1:1. YPV was a mixture of the individual single herbal granules. All of the above granules except Cordyceps sinesis were made from hot water extracts from the crude CHMs, and the quality of the CHMs complied with the standards of Chinese Pharmacopoeia. Voucher specimens of the above eleven herbs were deposited in the Herbarium of the School of Chinese Medicine, CUHK, with reference no. AR20221201-AR20221211, respectively. The granules were manufactured by Nong’s Company Limited complying with the good manufacturing practice (GMP) standards of the People’s Republic of China and Australia. Placebo granules were made from starch and dextrin, supplemented with food colorants and flavoring agents to mimic the appearance, smell, taste, and texture of YPV.

The quality control of YPV was conducted by Ultra High Performance Liquid Chromatography-Tandem Mass Spectrometry (UHPLC-MS). YPF granule (100 mg) was mixed with 1 mL of distilled water (containing 4 μg/mL mixture inner standards) and underwent a vortex oscillation for 1 min and a grinding for 2 min at 60 HZ and followed by a 60 min sonication in the ice water bath. After that the extract was centrifuged for 10 min at 12,000×*g* (4 ℃) and the supernatant was then fileted and diluted twice with distilled water (containing 4 μg/mL mixture inner standards) for the following UHPLC-MS analysis.

The UHPLC was performed using the Thermo-Obritrap-QE HF (ACQUITY UPLC HSS T3 100 mm × 2.1 mm, 1.8 um). The injected sample was 5 μL. The mobile phase A was water containing 0.1% formic acid and phase B was acetonitrile, with the flow rate at 0.35 mL/min and a gradient program as follows: 95% A at 0–2 min, 90–70% A at 2–4 min, 70–50% A at 4–8 min, 50–20% A at 8–10 min, 20–0% A at 10–14 min, 0% A at 14–15 min, 95% A at 15–16 min. For mass spectrometry, the spray voltages were at 3800 kV (positive) and 3200 kV (negative), the capillary temperature was 320 °C, the aux gas heater temperature was 350 ℃, the sheath gas flow rate was 35 Arb, the aux gas flow rate was 8 Arb, the mass range (m/z) was 100 to 1500, the NCE/stepped NCE was 10, 20, 40%.

Daily dosage of YPV or placebo granules were evenly divided into two packages at the Pharmacy of the Integrative Medical Center of HKIIM, CUHK, and the subjects were instructed to take one package each in the morning and the evening after meals, respectively. The granules were administered orally after dissolved in hot water and cool. The dosage was adjusted based on the age of each subject. The participants aged 13 or above took full dose, while those aged between 5 and 12 were given half dose. The participants were prohibited to take NSAIDs, antibiotics, anticoagulant agents, steroids, immunotherapy, antihistamines, probiotics, prebiotics, as well as any CM products used to treat AR in the whole study period.

### Assessments

A maximum of a 2-week run-in period was employed before randomization. Blood samples were collected at both screening visit and week 8 for determination of the renal and liver functions, and the complete blood picture (CBP). Eligible participants underwent medical assessment, with medical history, concomitant medication and vital signs evaluated and recorded by CMP investigators at baseline visit.

Total Nasal Symptom Score (TNSS), Rhinoconjunctivitis Quality of Life Questionnaire (RQLQ) (for participants aged 13 or above), or Paediatric Allergic Disease Quality of Life Questionnaire (PADQLQ) (for participants aged between 5 and 12), and Visual Analog Scale (VAS) for the frequency and severity of AR episodes were measured at baseline, weeks 4, 8, 12 and 16. The stool samples were collected with the cotton swab and stored immediately in the stool bottle with DNA stabilizing solution by the patients (within 24 h of the study visit day). The laboratory parameters and the subjects’ reported adverse events and/or serious adverse events were used to evaluate the safety. The assessment of adverse events was recorded based on CTCAE 4.0. All subjects were required to preserve their daily record to monitor their compliance and side effects of the study treatment. Besides, a 3-day food diary was obtained before taking stool specimens. Clinical assessments were performed in all follow-up visits according to the study schedule shown in our previous publication [22].

### Outcomes

The primary outcomes include the changes of the TNSS, RQLQ or PADQLQ at week 8 (the end of treatment). The secondary outcomes were (1) the changes of TNSS at weeks 4, 12 and 16; (2) the changes of the RQLQ or PADQLQ scores at weeks 4, 12 and 16; (3) the changes of frequency of AR episodes and their severity VAS at weeks 4, 8, 12 and 16; (4) the changes of the gut microbiota composition in stool samples at week 8; and (5) adverse events (AEs, graded by CTCAE) related to the study treatment.

### Determination of the gut microbiota of AR patients using 16S rRNA gene sequencing

Fresh fecal samples were harvested from AR patients and stored at −80 °C until use. The genomic DNA was extracted from fecal samples by Omega Mag-Bind Soil DNA Kit (M5635-02, Omega, USA) per the manufacturer’s protocols. DNA concentration was measured by Qubit 2.0 Fluorometer (Invitrogen, Life Technologies, CA), with 1.25 ng/μL being the minimum DNA concentration for sequencing. The hypervariable area of the 16S rRNA gene was amplified by polymerase chain reaction (PCR). The sequences analysis was conducted by UPARSE software package using the UPARSE-OTU and UPARSE-OTUref algorithms. The taxonomy of each sequence was performed by QIIME software and α-diversity indexes were compared using rarefed data. Principal coordinate analysis (PCoA) plot was conducted by R programming language.

### Declaration

The study will be conducted in compliance with the Declaration of Helsinki and Tokyo for humans.

### Statistical analysis

Sample size was calculated based on TNSS at the end of treatment. Detailed calculation was presented in the published protocol [23]. Data were expressed as mean ± standard deviation (SD). Intra-group comparisons between baseline and each visit were performed by paired t-test, and Wilcoxon signed rank test for non-parametric data. Analysis of covariance (ANCOVA) was used to examine if there were significant differences in the primary and secondary outcomes between two groups, with the accumulated hours of main rhinitis symptoms used as co-variables in the analysis. It is not uncommon to observe the missing values for one or more variables. The last observation carried forward (LOCF) was used to impute missing data. All 95% confidence intervals were two-sided, when *p* value was less than 0.05 considered as statistical significance. Data analyses were conducted using SPSS 29.0 version.

## Results

### Quality control of YPV

**Fig. 1:**
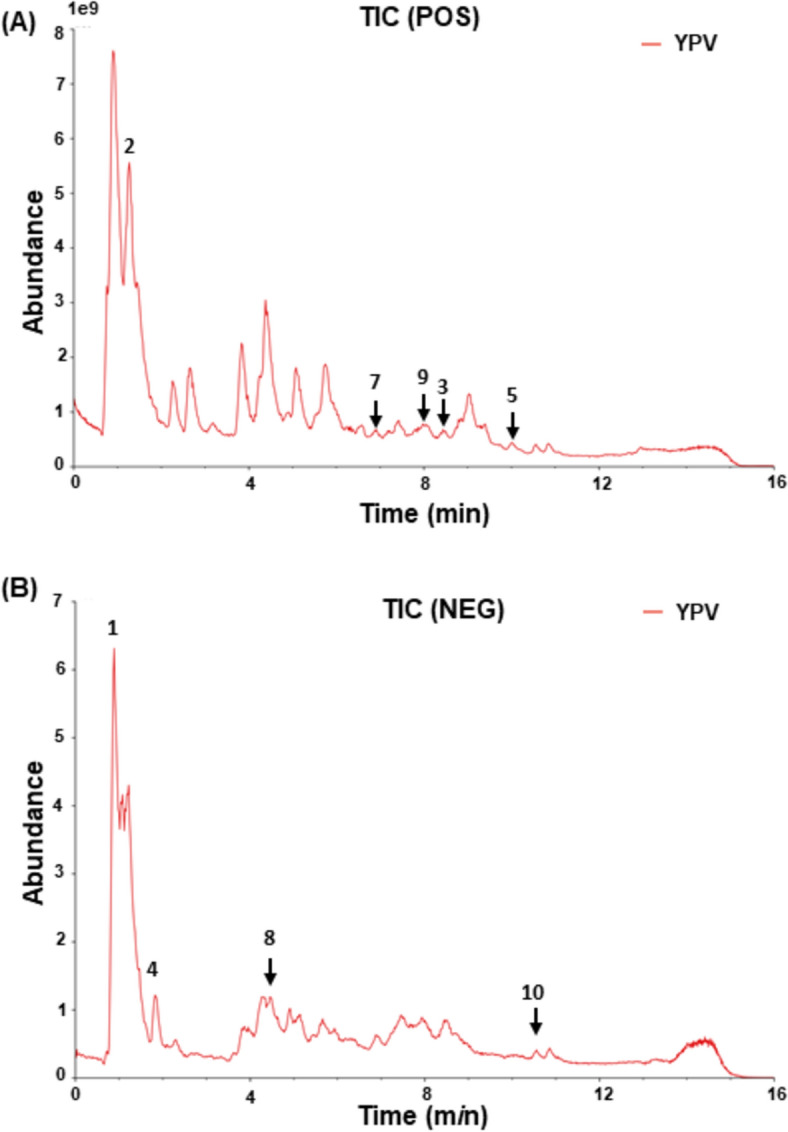
Total ion chromatogram of YPF granule. **A** Positive ion model and **B** negative ion model. The black numbers indicated the 10 identified compounds: (1) Citric acid, (2) Adenosine, (3) Magnolin, (4) α-Lactose, (5) Turanos, (6) L-arginine, (7) Soyasaponin Bb, (8) Amlaic acid, (9) Pinoresinol dimethyl ether and (10) Manninotriose

Based on the total ion chromatogram results in Fig. 1, the top ten components of YPF granule were identified (Table 1) as follows: in positive ion mode: (2) Adenosine (15.66%), (3) Magnolin (12.92%), (5) Turanos (10.35%), (6) L-arginine (6.99%), (7) Soyasaponin Bb (5.48%), (9) Pinoresinol dimethyl ether (4.83%) and in negative mode: (1) Citric acid (23.28%), (4) α-Lactose (10.69%), (8) Amlaic acid (5.24%), (10) Manninotriose (4.56%).

**Table 1:** Top ten chemical components in YPF granule

No	Name	Formula	RT (min)	Observed mass	Percentage (%)
1	Citric acid	C_6_H_8_O_7_	1.18	191.02	23.28
2	Adenosine	C_10_H_13_N_5_O_4_	1.27	268.10	15.66
3	Magnolin	C_23_H_28_O_7_	9.03	399.18	12.92
4	α-Lactose	C_12_H_22_O_11_	0.83	387.11	10.69
5	Turanose	C_12_H_22_O_11_	0.86	381.08	10.35
6	L-Arginine	C_6_H_14_N_4_O_2_	0.88	175.12	6.99
7	Soyasaponin Bb	C_48_H_78_O_18_	8.44	943.53	5.48
8	Amlaic acid	C_27_H_24_O_19_	4.35	633.07	5.24
9	Pinoresinol dimethyl ether	C_22_H_26_O_6_	8.84	369.17	4.83
10	Manninotriose	C_18_H_32_O_16_	0.83	549.17	4.56

### Participants

**Fig. 2:**
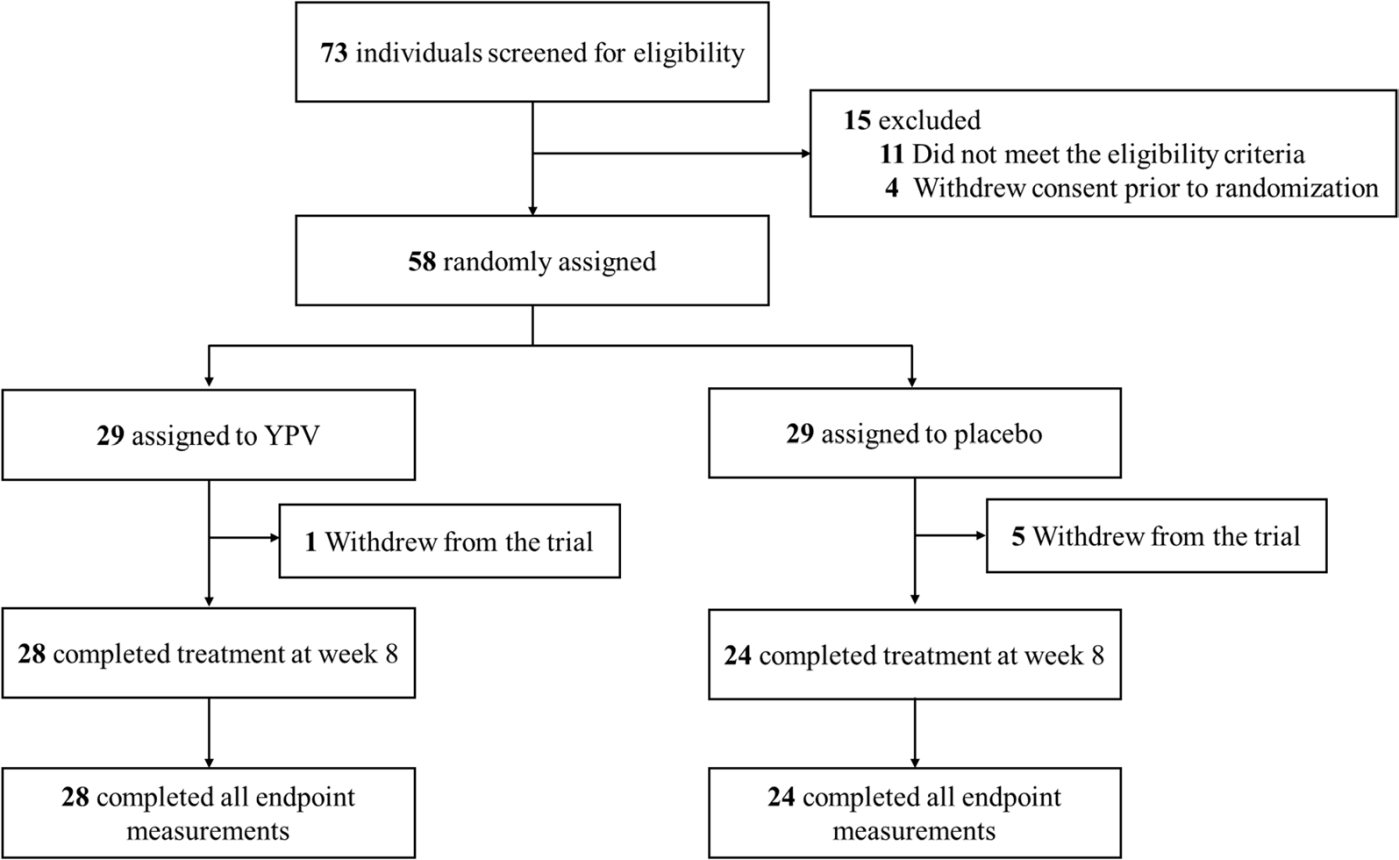
Clinical trial flow chart

A total of 73 participants were screened between 12 April 2022 and 20 June 2023 for eligibility. Among them, 58 participants were eligible and randomly assigned to receive either placebo (n = 29) or YPV (n = 29). Baseline characteristics, including baseline TNSS, VAS score, RQLQ score and PADQLQ score, were comparable between the two groups (Table 2). Six patients (1 from the YPV group due to loss of contact; 5 from the placebo group: 1 due to going aboard, 1 due to dizziness before blood collection, 2 because of personal issues, and 1 because of sore throat) withdrew their consent early after the randomization, leaving 96.6% (28 out of 29) patients in the YPV group and 82.8% (24 out of 29) patients in the placebo group completing the treatment at week 8. After the treatment, 28 participants in the YPV group and 24 participants in the placebo group completed the follow-up at week 16 (Fig. 2).

**Table 2:** Baseline characteristics of the participants

	YPV group (n = 29)	Placebo group (n = 29)
Age (years)	42.1 (17.6)	44.9 (18.6)
Sex		
Female	19 (65.5%)	13 (44.8%)
Male	10 (34.5%)	16 (55.2%)
BMI (kg/m^2^)	22.00 (3.08)	22.9 (3.71)
Chinese ethnicity	29 (100%)	29 (100%)
Non-Smoker	28 (96.6%)	28 (96.6%)
Ex-smoker	1 (3.45%)	1 (3.45%)
Accumulated hours of main rhinitis symptoms	9.72 (7.95%)	8.76 (6.99%)
History of AR (years)	16.46 (10.34)	17.97 (10.87)
TNSS at baseline	7.03 (1.59)	6.38 (2.21)
VAS score at baseline	5.84 (1.70)	5.93 (1.44)
RQLQ score at baseline^a^		
Activity limitations	4.06 (1.06)	3.21 (1.25)
Sleep impairment	2.60 (1.44)	2.32 (1.35)
Non-hay fever symptoms	2.60 (1.09)	2.30 (1.16)
Practical problems	3.83 (1.33)	3.16 (1.64)
Nasal symptoms	3.50 (1.04)	3.12 (1.08)
Eye symptoms	2.19 (1.25)	1.78 (1.21)
Emotional function	2.23 (1.39)	1.86 (1.32)
Total scores	2.91 (0.95)	2.47 (0.94)
PADQLQ score at baseline^b^		
Activities	2.00	18.00 (0.00)
Symptoms	14.00	37.00 (12.73)
Emotions	0.00	0.50 (0.71)
Total scores	16.00	55.50 (13.44)

Data were presented as mean (SD) or n (%)

^a^Data available on 56 participants aged 13 and above (YPV, n = 28; Placebo, n = 27)

^b^Data available on 3 participants aged 5–12 (YPV, n = 1; Placebo, n = 2)

### Primary and secondary outcomes

**Fig. 3:**
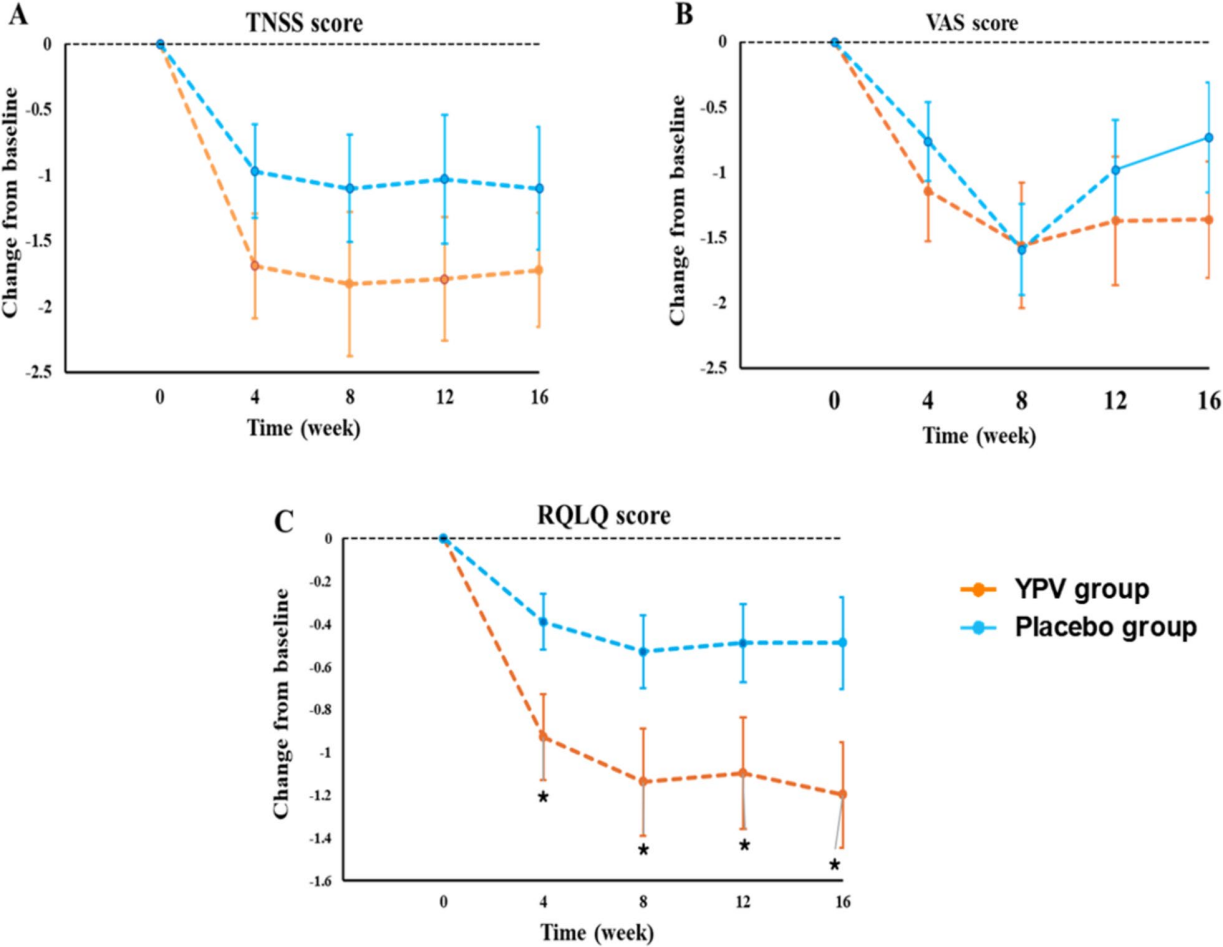
Effects of the YPV on the changes of primary and secondary outcomes from baseline during the study period. **A** TNSS score; **B** VAS score; **C** RQLQ score. Data were expressed as mean (SD). *p < 0.05 compared with the placebo group

The changes of the primary and secondary outcomes from baseline were shown in Table 3 and Fig. 3. We found that YPV treatment markedly improved the RQLQ score in AR patients at weeks 4, 8, 12, and 16 (between-group difference, 0.69 [95% CI, −0.001 to 2.49], *p* = 0.05 for week 5; 0.92 [95% CI, 0.03 to 1.82], *p* = 0.04 for week 8; 0.99 [95% CI, 0.03 to 1.95], *p* = 0.04 for week 12; 1.11 [95% CI, 0.13 to 2.08], *p* = 0.03 for week 16) when compared with the placebo group. Moreover, as shown in Table 4, the YPV treatment markedly improved the RQLQ total score and its individual symptom scores in AR patients at weeks 4, 8, 12, and 16 (*p* < 0.01 for all), when compared with those at the baseline. One the other hand, placebo also significantly decreased the RQLQ total score at weeks 4, 8, and 12 (*p* < 0.05 for all), as compared with those at the baseline.

**Table 3:** The changes of primary and secondary outcomes from baseline during the study period (data presented as mean (SD))

Outcomes	Follow-up duration	YPV group	Placebo group	Adjusted between-group difference, mean (95% CI)	*p* value	Score Reference
TNSS score		N = 29	N = 29			0 = None (No symptoms evident)
	Week 4	−1.69 (2.16)	−0.97 (1.92)	0.92 (−0.69, 2.52)	0.26	1 = Mild (Symptom present but easily tolerated)
	Week 8	−1.83 (2.96)	−1.10 (2.21)	0.82 (−1.26, 2.89)	0.43	2 = Moderate (Definite awareness of symptom; bothersome but tolerable)
	Week 12	−1.79 (2.53)	−1.03 (2.64)	1.44 (−0.72, 3.61)	0.19	3 = Severe (Symptom hard to tolerate; interferes with daily activity)
	Week 16	−1.72 (2.33)	−1.10 (2.51)	0.61 (−1.42, 2.63)	0.55	
RQLQ score		N = 28	N = 27			0 = Not troubled
	Week 4	−0.93 (1.07)	−0.39 (0.68)	0.69 (−0.001, 1.38)	0.05	1 = Hardy troubled at all
	Week 8	−1.14 (1.33)	−0.53 (0.89)	0.92 (0.03, 1.82)	0.04*	2 = Somewhat troubled
	Week 12	−1.10 (1.38)	−0.49 (0.95)	0.99 (0.03, 1.95)	0.04*	3 = Moderated troubled
	Week 16	−1.20 (1.31)	−0.49 (1.12)	1.11 (0.13, 2.08)	0.03*	4 = Quite a bit troubled 5 = Very troubled 6 = Extremely troubled
VAS score		N = 29	N = 29			1—Excellent
	Week 4	−1.14 (2.08)	−0.76 (1.63)	0.96 (−0.58, 2.49)	0.22	2—Very good
	Week 8	–1.56 (2.59)	−1.59 (1.88)	1.13 (–0.72, 2.97)	0.23	3—Good
	Week 12	–1.37 (2.66)	–0.98 (2.05)	1.85 (–0.11, 3.80)	0.06	4—Fair
	Week 16	–1.36 (2.4)	–0.73 (2.26)	1.76 (–0.18, 3.69)	0.08	5—Poor 6—Terrible

Data were expressed as mean (SD)

*p < 0.05 compared with the placebo group

**Table 4:** The changes of RQLQ and individual symptom scores from baseline during the study period

Changes from baseline	Follow-up duration	YPV group	Placebo group
RQLQ total scores		N = 28	N = 27
	Week 4	−0.97 (1.08) ***	−0.42 (0.70) *
	Week 8	−1.18 (1.34) ***	−0.56 (0.91) *
	Week 12	−1.14 (1.39) ***	−0.50 (0.98) *
	Week 16	−1.24 (1.31) ***	−0.51 (1.16)
Activity		N = 28	N = 27
	Week 4	−1.38 (1.43) ***	−0.61 (1.23) *
	Week 8	−1.67 (1.64) ***	−0.76 (1.25) *
	Week 12	−1.53 (2.06) ***	−0.67 (1.39)
	Week 16	−1.67 (1.67) ***	−0.92 (1.48) **
Sleep quality		N = 28	N = 27
	Week 4	−0.95 (1.46) ***	−0.14 (0.87)
	Week 8	−1.31 (1.69) ***	−0.54 (1.12)
	Week 12	−0.78 (1.59) **	−0.28 (1.41)
	Week 16	−1.18 (1.37) ***	−0.17 (1.55)
Non-eye/nasal symptoms		N = 28	N = 27
	Week 4	−0.75 (1.35) **	−0.20 (0.97)
	Week 8	−1.02 (1.53) ***	−0.27 (1.13)
	Week 12	−1.14 (1.56) ***	−0.38 (1.16)
	Week 16	−1.15 (1.49) ***	−0.40 (1.33)
Practical problems		N = 28	N = 27
	Week 4	−1.27 (1.38) ***	−0.74 (0.98) **
	Week 8	−1.48 (1.62) ***	−0.94 (1.27) **
	Week 12	−1.44 (1.58) ***	−0.99 (1.31) **
	Week 16	−1.47 (1.77) ***	−0.86 (1.58) *
Nasal symptoms		N = 28	N = 27
	Week 4	−1.26 (1.36) ***	−0.78 (0.97) **
	Week 8	−1.41 (1.44) ***	−0.79 (1.25) **
	Week 12	−1.30 (1.50) ***	−0.74 (1.25) *
	Week 16	−1.37 (1.47) ***	−0.74 (1.35) *
Eye symptoms		N = 28	N = 27
	Week 4	−0.81 (0.95) ***	−0.51 (0.93) *
	Week 8	−0.78 (1.44) **	−0.51 (0.85) *
	Week 12	−0.99 (1.29) ***	−0.51 (1.07) *
	Week 16	−0.96 (1.47) **	−0.33 (1.25)
Emotional function		N = 28	N = 27
	Week 4	−0.69 (1.24) **	−0.18 (0.92)
	Week 8	−0.97 (1.42) **	−0.46 (1.38)
	Week 12	−0.89 (1.43) **	−0.18 (1.08)
	Week 16	−1.11 (1.40) ***	−0.32 (1.31)

Data were expressed as mean (SD) for RQLQ and individual symptoms

*p < 0.05, **p < 0.01, ***p < 0.001 for changes in the symptom scores from the baseline within groups

However, oral administration of the YPV granules had no significant effect on TNSS score (between-group difference, 0.92 [95% CI, −0.69 to 2.52], *p* = 0.26 at week 4, 0.82 [95% CI, −1.26 to 2.89], *p* = 0.43 at week 8, 1.44 [95% CI, −0.72 to 3.61], *p* = 0.43 at week 12, 0.61 [95% CI, −1.42 to 2.63], *p* = 0.55 at week 16) of the AR patients, when compared with the placebo group. On the other hand, as shown in Table 5, YPV treatment could significantly improve the TNSS and its component scores in AR patients at weeks 4, 8, 12, and 16 when compared with those at the baseline (*p* < 0.05 for all). AR patients could benefit more from the YPV than the placebo at weeks 4, 8, 12, and 16, as evidenced by greater reduction of TNSS and most of the component scores from the baseline for each time point in the YPV treatment group.

**Table 5:** The changes of TNSS and individual symptom scores from baseline during the study period

Changes from baseline	Follow-up duration	YPV group	Placebo group
TNSS total scores		N = 29	N = 29
	Week 4	−1.75 (2.17) ***	−1.08 (2.08) *
	Week 8	−1.89 (3.00) ***	−1.08 (2.34)
	Week 12	−1.86 (2.55) ***	−1.00 (2.83)
	Week 16	−1.79 (2.35) ***	−1.08 (2.69) *
Nasal congestion		N = 29	N = 29
	Week 4	−1.89 (0.69) *	−1.43 (0.69)
	Week 8	−1.5 (0.69) *	−1.38 (0.50)
	Week 12	−1.46 (0.84) *	−1.38 (0.77)
	Week 16	−1.54 (0.69) *	−1.25 (0.68) *
Sneezing		N = 29	N = 29
	Week 4	−1.29 (0.71) *	−1.1 (0.76) ***
	Week 8	−1.14 (0.76) *	−1.08 (0.56) **
	Week 12	−1.21 (0.74) **	−1.17 (0.82)
	Week 16	−1.21 (0.63) *	−1.29 (0.75)
Rhinorrhea		N = 29	N = 29
	Week 4	−1.32 (0.91) ***	−1.39 (0.63)
	Week 8	−1.29 (0.81) **	−1.38 (0.70)
	Week 12	−1.36 (0.83) **	−1.54 (0.72)
	Week 16	−1.36 (0.73) **	−1.38 (0.88)
Nasal itching		N = 29	N = 29
	Week 4	−1.21 (0.74) *	−1.32 (0.86)
	Week 8	−1.18 (0.86) *	−1.31 (0.74)
	Week 12	−1.11 (0.83) **	−1.13 (0.74)
	Week 16	−1.11 (0.74) ***	−1.21 (0.88)

Data were expressed as mean (SD) for TNSS and individual symptoms

*p < 0.05, **p < 0.01, ***p < 0.001 for change in symptom score from baseline within groups

No significant difference was found in the changes of VAS score from the baseline at weeks 4, 8, 12, and 16 between the YPV group and the placebo group (between-group difference, 0.96 [95% CI, −0.58 to 2.49], *p* = 0.22 for week 4; 1.13 [95% CI, −0.72 to 2.97], *p* = 0.23 for week 8; 1.85 [95% CI, −0.11 to 3.80], *p* = 0.06 for week 12; 1.76 [95% CI, −0. 18 to 3.69], *p* = 0.08 for week 16), while the TNSS and VAS scores were reduced significantly at week 8 from baseline in both groups. As shown in Table 6, the YPV treatment significantly reduced the VAS score in AR patients at weeks 4, 8, 12, and 16 when compared with those at the baseline (*p* < 0.01 for all), while placebo also markedly decreased the VAS score at weeks 4 (*p* < 0.05) and 8 (*p* < 0.01) as compared with those at the baseline (*p* < 0.05). The decrease of VAS score from the baseline in the YPV group was more pronounced than that in the placebo group at weeks 4, 12, and 16.

**Table 6:** The changes of VAS score from baseline during the study period

Changes from baseline	Follow-up duration	YPV group	Placebo group
VAS		N = 29	N = 29
	Week 4	−1.18 (2.11) **	−0.81 (1.74) *
	Week 8	−1.61 (2.62) ***	−1.64 (1.9) **
	Week 12	−1.42 (2.69) **	−0.91 (2.1)
	Week 16	−1.4 (2.43) **	−0.6 (2.32)

Data were expressed as mean (SD) for VAS scores

*p < 0.05, **p < 0.01, ***p < 0.001 for change in VAS score from baseline within groups

The PADQLQ, a questionnaire, was designed for determination the quality of life for children with AR who were aged 5–12. In this clinical trial, only 3 participants aged 5–12 were included, including 1 participant in the YPV group and 2 participants in the placebo group. Data analysis was deemed inappropriate for such a small sample size.

### Gut microbiota composition in stools at week 8

**Fig. 4:**
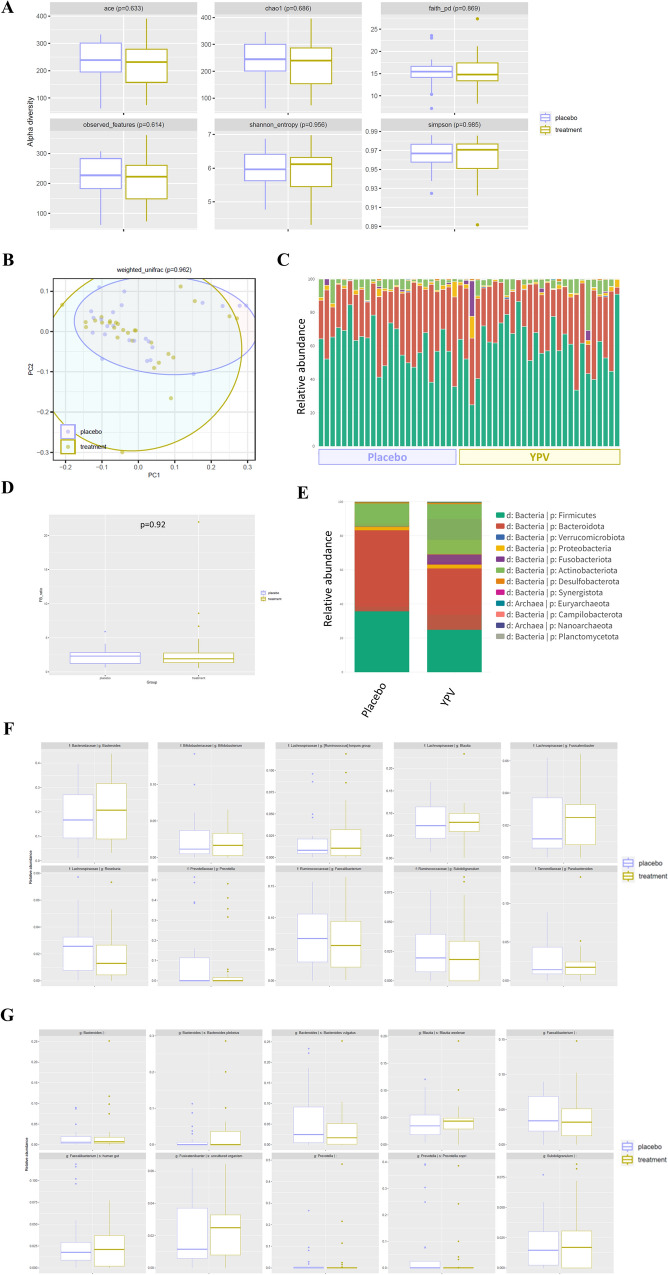
Effects of the YPV on gut dysbiosis in AR patients. **A** Effects of the YPV on α-diversity in AR patients, including ACE, chao1, Faith’s PD, observed features, Shannon’s entropy, and Simpson index; **B** PCoA of microbial community structure; **C** Gut microbiota composition at the phylum level; **D** Firmicutes/Bacteroidota ratio; **E** The relative abundance of gut microbiota composition between two groups at the phylum level; **F** Gut microbiota composition at the genus level; **G** Gut microbiota composition at the species level

As shown in Fig. 4A, α-diversity analysis results showed no significant differences in the abundance-based coverage estimator (ACE) (*p* = 0.633), chao1 (*p* = 0.686), Faith’s phylogenetic diversity (Faith’s PD) (*p* = 0.869), observed features (*p* = 0.614), Shannon’s entropy (*p* = 0.956), and Simpson index (*p* = 0.985) between the YPV group and the placebo group, indicating that there were no significant differences in sequencing depth index and bacterial richness between these two groups. As shown in Fig. 4B, the PCoA results suggested that microbial communities in patients with the treatment of YPV were not clustered away and differed from those in patients treated with placebo (*p* = 0.962, Weighted Unifrac), indicating that the gut microbial community composition was not altered after drug treatments. Then the relative abundance of gut microbiota distributions was identified in the stool samples of AR patients. At the phylum level, the gut microbiota in patients with AR primarily comprised *Firmicutes*, *Bacteroidota*, *Proteobacteria*, and *Actinobacteria* (Fig. 4C, E). No significant difference in *Firmicutes*/*Bacteroidota* (F/B) ratio was found between the YPV group and the placebo group at week 8 (*p* = 0.92, Fig. 4D). There were no statistical differences in the relative abundance of gut microbiota composition at the genus level and the species level between the YPV group and the placebo group (Fig. 4F, G).

### Adverse events (AEs)

The reported AEs were summarized in Table 7. There were no serious AEs or withdrawals due to AEs. No significant differences in the occurrence of AEs were found between the YPV group and the placebo group. The most frequent AEs was gastrointestinal discomfort, and reported by 26.7, 50.0, 18.2, and 23.1% participants at weeks 4, 8, 12, and 16, respectively. Most AEs were mild and recovered without further medical intervention.

**Table 7:** Adverse events

	YPV group (n = 28)	Placebo group (n = 24)
Week 4		
Gastrointestinal disorders	5 (17.6%)	3 (12.5%)
Nervous system disorders	3 (10.7%)	4 (16.7%)
Musculoskeletal and connective tissue disorders	2 (7.1%)	2 (8.3%)
Infections and infestations	2 (7.1%)	1 (4.2%)
Skin and subcutaneous tissue disorders	2 (7.1%)	1 (4.2%)
Cardiac disorders	0	1 (4.2%)
General disorders and administration site conditions	1 (3.6%)	0
Renal and urinary disorders	0	1 (4.2%)
Reproductive system and breast disorders	1 (3.6%)	0
Respiratory, thoracic and mediastinal disorders	0	1 (4.2%)
Total	16 (57.1%)	14 (58.3%)
Week 8		
Gastrointestinal disorders	7 (25%)	5 (20.8%)
Infections and infestations	0	4 (16.7%)
Ear and labyrinth disorders	2 (7.1%)	0
Respiratory, thoracic and mediastinal disorders	1 (3.6%)	1 (4.2%)
Injury, poisoning and procedural complications	0	1 (4.2%)
Nervous system disorders	1 (3.6%)	0
Renal and urinary disorders	0	1 (4.2%)
Reproductive system and breast disorders	1 (3.6%)	0
Total	12 (42.9%)	12 (50.0%)
Week 12		
Gastrointestinal disorders	1 (3.6%)	1 (4.2%)
General disorders and administration site conditions	2 (7.1%)	0
Immune system disorders	0	1 (4.2%)
Infections and infestations	0	1 (4.2%)
Injury, poisoning and procedural complications	0	1 (4.2%)
Musculoskeletal and connective tissue disorders	1 (3.6%)	0
Nervous system disorders	1 (3.6%)	0
Psychiatric disorders	0	1 (4.2%)
Investigating	1 (3.6%)	0
Total	6 (21.4%)	5 (20.8%)
Week 16		
Gastrointestinal disorders	2 (7.1%)	1 (4.2%)
Nervous system disorders	2 (7.1%)	1 (4.2%)
Infections and infestations	1 (3.6%)	1 (4.2%)
Musculoskeletal and connective tissue disorders	1 (3.6%)	1 (4.2%)
General disorders and administration site conditions	1 (3.6%)	0
Respiratory, thoracic and mediastinal disorders	0	1 (4.2%)
Skin and subcutaneous tissue disorders	1 (3.6%)	0
Total	8 (28.6%)	5 (20.8%)

## Discussion

The trial results indicated that treatment with YPV produced greater improvement in the quality of life domain of AR patients than placebo, and this effect could last for two months after the treatment. However, no evidence was shown on its clinical effects on topical symptoms of AR as evaluated by TNSS and VAS score.

The use of CM in the treatment of AR is widely accepted in the Chinese communities, and is also gaining reputation in other parts of the world [24]. CM is distinguished by its individualized treatment by classifying a given disease into different syndrome patterns, thereby requiring different treatment regimens. In AR, the common syndrome patterns include: (1) Deficiency in the lung with pathogenic cold; (2) Qi deficiency in the spleen; (3) Yang deficiency in the kidney; and (4) Stagnant heat in the lung [25]. The clinical manifestations of the first three patterns signify a Yin pattern which is characterized by a lack of energy, while the last pattern (Stagnant heat) signifies a Yang pattern with excess in energy. In clinical reality, most patients with AR possess Yin pattern over Yang pattern, especially in school children [12, 26]. In this regard, YPV was designed to alleviate the symptoms of AR by balancing the Yin pattern which involves strengthening the lung and spleen Qi and expelling pathogenic cold. YPV consists of the original Yu-Ping-Feng Powder that focuses on strengthening the lung and spleen Qi, together with a number of CM to nourish the kidney Qi and expel pathogenic cold. Therefore, YPV is a tailored formula for the treatment of AR with the syndrome type of lung and spleen Qi deficiency. Previous studies have shown satisfactory results of Yu-Ping-Feng Powder or its variations in the treatment of patients with AR. However, syndrome patterns of the subjects were often not identified, or the trials were only conducted in a selected group instead of the general population [10, 11, 13]. Our trial was designed to recruit AR patients from general public in Hong Kong, and our CMPs were available to determine the participants’ syndrome patterns during the screening visits, which could guarantee the accurate selection of participants according to syndrome pattern, and maximize the benefit of YPV on AR patients according to CM theory.

In this study, YPV was found to have effects on improving the overall quality of life of the AR patients, while its effects on the specific symptoms of AR were not significant. The clinical evaluation on the prognosis of AR predominantly consists of subjective assessment [27, 28], including AR symptom scoring and quality of life questionnaire. The symptoms of AR patients are widely according to the scoring of four nasal symptoms, i.e., sneezing, rhinorrhea, nasal itching, and nasal congestion, and/or some ocular symptoms such as ocular itchy/sensation of foreign body/redness of the eyes and tearing [28]. Two widely used scales, TNSS and VAS, were self-assessed by the participants in our trial to evaluate the severity of AR symptoms.

A trend towards improvement in TNSS and VAS scores was observed with the YPV treatment, when compared with the placebo group. However, there were no statistical differences between these two groups at each time point. It may be attributed to two factors: first and probably the most important one, the sample size of this trial (58 participants in total) was not large enough to detect the treatment effect; second, the duration of the treatment period probably was not long enough to cause sufficient treatment effect. Apart from nasal or ocular symptoms, AR also exerts a negative impact on patients’ quality of life, including work efficiency, academic performance, social life, and mental well-being, making it a serious public health concern [29, 30]. In this study, we utilized the RQLQ [31], a widely accepted and validated tool, to assess the quality of life in adult patients with AR. Our results demonstrated that AR patients benefited more from the YPV treatment than placebo in the domain of quality of life, particularly in activity levels, sleep quality, and emotional well-being. Further exploration on YPV with different treatment duration and larger sample size is warranted.

Recent evidence suggests that alteration of gut microbiota composition may be related to AR pathology [32]. Previous studies found that the gut microbiota composition in AR patients differed from that of healthy controls, with a higher level of *Bacteroidetes* abundance and decreased relative abundance of *Actinobacteria*, *Clostridiales*, and *Proteobacteria* in AR patients [33–35]. The potential benefits of probiotics in the prevention and treatment of various diseases, including AR, have increased interest worldwide in recent years. Although several clinical studies reported the therapeutic potentials of single probiotic strain or mixed probiotic strains on AR [36–38], the anti-AR effects of these probiotic supplements have yet to be confirmed due to a lack of sufficient evidence. Moreover, the association between the gut microbiota and the AR progression or the severity of AR symptoms has not been established. The present study explored the gut microbiota diversity and composition between the YPV group and the placebo group using 16S rRNA gene sequencing. Our results found no significant differences in the gut microbiota diversity and composition between these two groups, indicating that the therapeutic effects of the YPV on AR were unlikely associated with gut microbiota.

In addition to the clinical efficacy, our study also showed a satisfactory safety profile associated with the YPV treatment. Although a number of participants in both groups reported at least one AE, these reported AEs were mostly mild and did not lead to treatment discontinuation or withdrawal during the trial. Furthermore, no significant differences in the occurrence of AEs were found between the YPV group and the placebo group, revealing that YPV treatment is well-tolerated and safe.

Some limitations should be acknowledged for this study. Firstly, as stated previously, the sample size in this clinical trial was relatively small, which may have limited the trial’s ability to detect statistically significant improvements in efficacy with YPV treatment. Secondly, all participants in this study were enrolled from Hong Kong, China. Further studies with different ethnicities and genetic backgrounds will be required. Thirdly, the durability of the intervention has not been fully investigated due to the relatively short follow-up period.

In conclusion, in this randomized, double-blind, and placebo-controlled clinical trial, the YPV, an empirical herbal formula commonly used in CM clinic practice in Hong Kong for AR, has been found to be well-tolerated and exert a beneficial and lasting effect on improving the quality of life for patients with AR. However, no evidence was found on its effect on topical symptoms.

## Data Availability

All data generated or analyzed during the present study are included in this published article.

## Abbreviations

AR: Allergic rhinitis
AEs: Adverse events
CBP: Complete blood picture
CHMs: Chinese herbal medicines
CM: Chinese medicine
CMP: Chinese Medicine Practitioners
GMP: Good manufacturing practice
IgE: Immunoglobulin E
IL-4: Interleukin-4
LOCF: Last observation carried forward
NSAIDs: Nonsteroidal anti-inflammatory drugs
PADQLQ: Paediatric Allergic Disease Quality of Life Questionnaire
PCoA: Principal coordinate analysis
PCR: Polymerase chain reaction
RQLQ: Rhinoconjunctivitis Quality of Life Questionnaire
SD: Standard deviation
TCM: Traditional Chinese medicine
TNSS: Total Nasal Symptom Score
VAS: Visual Analog Scale
YPV: Yu-Ping-Feng Powder with Variation
